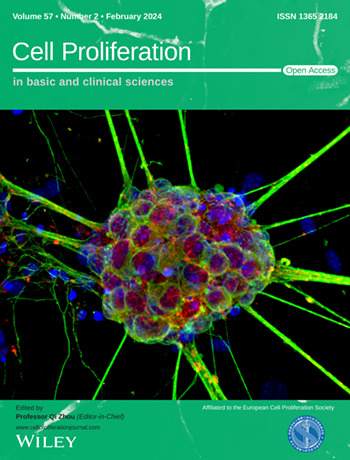# Featured Cover

**DOI:** 10.1111/cpr.13615

**Published:** 2024-02-07

**Authors:** Zihui Xu, Yanan Guo, Kangjian Xiang, Dongchang Xiao, Mengqing Xiang

## Abstract

The cover image is based on the Original Article *Rapid and efficient generation of a transplantable population of functional retinal ganglion cells from fibroblasts* by Zihui Xu et al., https://doi.org/10.1111/cpr.13550.